# Ginsenoside Ro ameliorates cognitive impairment and neuroinflammation in APP/PS1 mice via the IBA1/GFAP-MAPK signaling pathway

**DOI:** 10.3389/fphar.2025.1528590

**Published:** 2025-02-24

**Authors:** Tianyao Li, Jiaxin Chen, Zhouyuan Xie, Jiansong Fang, Qiqing Wu, Xinyue Cao, Ziying Chen, Yiyun Wang, Qiqi Fan, Qi Wang, Jinman Liu

**Affiliations:** ^1^ Science and Technology Innovation Center, Guangzhou University of Chinese Medicine, Guangzhou, China; ^2^ Affiliated Jiangmen TCM Hospital of Ji’nan University, Jiangmen, China

**Keywords:** Alzheimer’s disease, APP/PS1 mice, Ginsenoside Ro, neuronal apoptosis, neuroinflammation, IBA1/GFAP-MAPK pathway

## Abstract

**Introduction:**

Ginseng, known as the “king of herbs,” has long been used in traditional Chinese medicine due to its beneficial properties, including anti-aging, anti-inflammatory, and anti-apoptotic effects. Ginsenosides, the active compounds in ginseng, have shown promise in treating neurodegenerative diseases such as Alzheimer’s disease (AD). This study investigates the therapeutic potential of Ginsenoside Ro and its underlying mechanisms in AD treatment.

**Methods:**

In this study, male APP/PS1 transgenic mice were divided into five groups and treated with Ginsenoside Ro or ginseng for one month. Cognitive function and anxiety were assessed through behavioral tests, including the open field test (OFT) and Morris water maze (MWM). To evaluate Aβ deposition, neuronal apoptosis, neuroinflammation, and the MAPK pathway, various techniques were employed: Thioflavin-T staining, Nissl staining, immunofluorescence, Western blot, and qRT-PCR analyses.

**Results:**

Ginsenoside Ro significantly improved cognitive function and reduced anxiety in APP/PS1 mice. It also decreased Aβ deposition and ameliorated neuronal apoptosis in the cerebral cortex. The treatment regulated the expression of pro-apoptotic proteins (Bax and Caspase3) and increased the anti-apoptotic protein Bcl-2. Additionally, Ginsenoside Ro reduced neuroinflammation by decreasing IBA1-positive microglia and GFAP-positive astrocytes and lowering pro-inflammatory cytokines while enhancing anti-inflammatory cytokine IL-10. Furthermore, the phosphorylation levels of p38 and JNK in the MAPK pathway were significantly reduced, suggesting a key mechanism for its therapeutic effects.

**Discussion:**

These findings provide strong evidence supporting Ginsenoside Ro as a potential therapeutic agent for Alzheimer’s disease. Its effects appear to be mediated through the modulation of the IBA1/GFAP-MAPK pathway, which may offer new insights into AD treatment strategies.

## Graphical Abstract

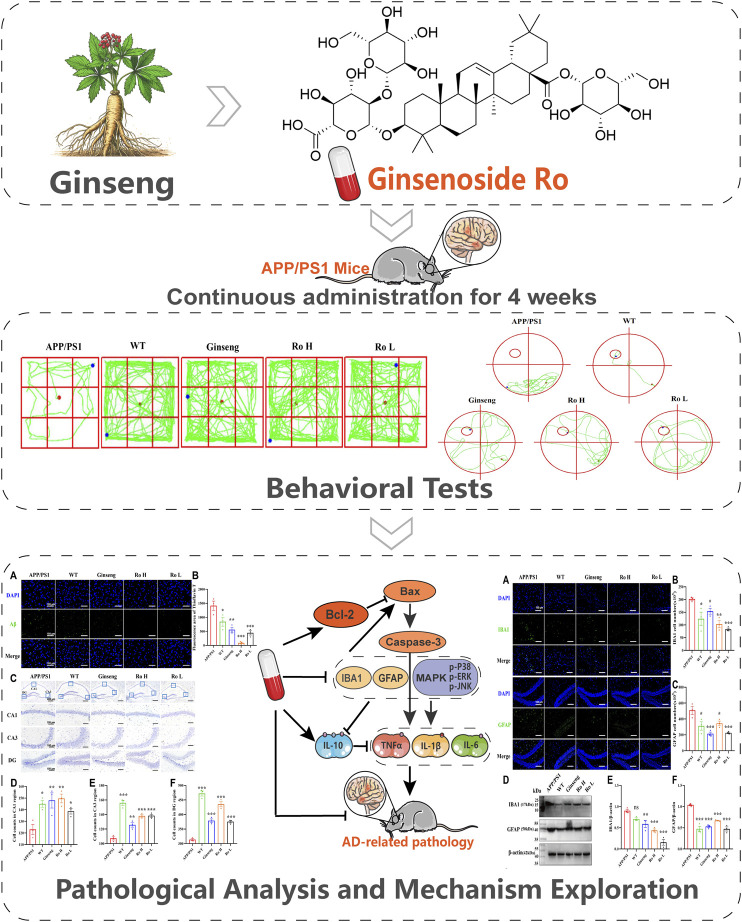

## 1 Introduction

Alzheimer’s disease (AD) is a neurodegenerative disorder characterized by memory loss and cognitive decline (Zhang et al., 2023). With an aging population, the need for effective drugs to treat AD has become increasingly urgent. The etiology of AD is not fully understood, but known factors include Aβ amyloid plaque deposition, neuroinflammation, tau protein hyperphosphorylation, oxidative stress, endoplasmic reticulum stress, mitochondrial dysfunction, and autophagy (Chen and Zhang, 2022). These factors reinforce each other, creating a vicious cycle that ultimately leads to neuronal death and the progression of AD (Gavrilova, 2022).

β-amyloid (Aβ) is a core component of neuroinflammatory plaques and is a protein hydrolysis product of amyloid β precursor protein (APP) (Gao et al., 2022). Aβ-induced neuroinflammation and neuronal apoptosis accelerate AD pathogenesis (Zhang et al., 2019). Since 1992, the amyloid cascade hypothesis has been crucial in explaining AD’s etiology and pathogenesis (Hardy and Higgins, 1992). Apoptosis, a form of programmed cell death, plays a significant role in various physiological and pathological processes. In AD, extensive neuronal loss is primarily due to apoptosis (Xing D. et al., 2024). Aβ exhibits dual pathological effects: direct neuronal damage and the induction of neuroinflammation. Therefore, therapeutic agents that can simultaneously modulate neuronal apoptosis and attenuate neuroinflammation triggered by Aβ neurotoxicity represent promising candidates for treating AD (Wang et al., 2020).

MAPKs (mitogen-activated protein kinases) are serine/threonine protein kinases involved in signaling pathways that regulate cellular functions in response to extracellular stimuli (Chakraborty et al., 2023). In mammalian cells, there are three types of MAPKs: extracellular signal-regulated kinase (ERK), N-terminal kinase (JNK), and p38. p-p38 is mainly activated by pro-inflammatory cytokines and environmental stresses (Chakraborty et al., 2023; Yue and López, 2020). p-p38 accumulation through kinase cascades is a pathological feature of AD. Phosphorylated p38 is significantly upregulated in conditions like chronic inflammation, triggering downstream signaling and leading to pathological deterioration (Falcicchia et al., 2020). Reducing phosphorylated p38 levels has been shown to lower microglia and astrocyte activation and Aβ deposition in AD mice (Son et al., 2023). Thus, decreasing p-p38 expression and its downstream signaling can reduce microglia and astrocyte activation and Aβ deposition, improving neuronal apoptosis and neuroinflammation.

Due to the incomplete understanding of AD pathogenesis, many current clinical drugs are ineffective or have serious side effects. In recent years, Traditional Chinese Medicine (TCM) has played an increasingly important role in treating various diseases (Xing et al., 2024b; Xing et al., 2024c). Ginseng (*Panax ginseng* C. A. Mey. root), known as the “king of herbs,” is a perennial herb with anti-aging, anti-inflammatory, and anti-apoptotic effects (Zhou et al., 2023). Numerous studies have shown that ginseng and ginsenosides are effective in treating neurodegenerative diseases (de Oliveira Zanuso et al., 2022; Manju, 2024; Feng et al., 2022; Zhou et al., 2024). Various ginsenosides have demonstrated significant clinical efficacy in improving AD (Joo and Lee, 2005; Jiao and Jia, 2022; Liu et al., 2024a). Through network pharmacology, four ginsenosides related to AD pathogenesis were pre-screened, and Ginsenoside Ro showed good therapeutic efficacy *in vitro* (Liu et al., 2023a), However, the exact mechanism remains unclear, necessitating further validation of Ginsenoside Ro’s molecular mechanisms in treating AD using APP/PS1 mice.

This study investigated the therapeutic mechanism of Ginsenoside Ro on AD using APP/PS1 mice. The results demonstrated that Ginsenoside Ro reduced the activation levels of microglia and astrocytes by down-regulating the expression of p-p38, p-ERK, and p-JNK. This downregulation decreased Aβ deposition and ameliorated neuroinflammation and neuronal apoptosis, thereby preventing AD progression.

## 2 Materials and methods

### 2.1 Reagents

Ginsenoside Ro (E-0180, Purity: 98%) was purchased from Shanghai Tauto Biotech. Ginseng (batch no. 2403035) was obtained from Guangzhou Jizhi Bao Chinese Medicine Co. and authenticated by Professor Wei Li (School of Chinese Materia Medica, Guangzhou University of Chinese Medicine). All samples were stored at Science and Technology Innovation Center, Guangzhou University of Chinese Medicine. Thioflavin-T (T168914) was procured from Aladdin. Nissl Staining Solution (C0117) and Antifade Mounting Medium with 4′,6-Diamidino-2-Phenylindole (DAPI) (P0131) were obtained from Beyotime Co., Ltd. IL-1β ELISA kit(MM-0040M2), IL-6 ELISA kit(MM-0163M2), TNFα ELISA kit(MM-0132M2) were obtained from MMBio Co., Ltd. TUNEL assay kit (GDP1042) were purchased from servicebio Co., Ltd. β-Actin Rabbit Antibody (AF7018), Bax Rabbit antibody (AF0120), Bcl-2 Rabbit antibody (AF6139), and Caspase 3 Rabbit antibody (AF6311) were purchased from Affinity Biosciences LTD. IBA1 Rabbit mAb (A19776) was obtained from ABclonal Technology Co., Ltd. GFAP Rabbit Antibody (PAB32097) was purchased from Bioswamp Co., Ltd. p-P38 Rabbit Antibody (PC2210) was obtained from Abmart Technology Co., Ltd. P38 Rabbit Antibody (ET1602-26),ERK Rabbit Antibody (ET1601-29),JNK Rabbit Antibody (ET1601-28),p-ERK Rabbit Antibody (ET1601-13) and p-JNK Rabbit Antibody (ET1601-42) were purchased from HuaBio Co., Ltd. FITC-labeled Goat Anti-Mouse (A0568) and FITC-labeled Goat Anti-Rabbit IgG were procured from Beyotime Co., Ltd. Evo M-MLV Reagent Premix (for qPCR) and Evo M-MLV Reverse Transcription Kit (for qPCR) were obtained from Accurate Biology Co., Ltd. BCA Protein Quantification Kit (20201ES76), PAGE Gel Quick Preparation Kit (12.5%) (20326ES62), PAGE Gel Quick Preparation Kit (10%) (20325ES62), and 5**×**SDS-PAGE Protein Loading Buffer (20315ES05) were purchased from Yeasen Biotechnology (Shanghai) Co., Ltd. Protease inhibitor cocktail for general use, 100X (P1005), and RIPA Lysis Buffer (P0013K) were procured from Beyotime Co., Ltd. PVDF membranes (IPVH00010, ISEQ00010) were purchased from Shanghai Morhan Biotechnology Co.

### 2.2 Animal grouping and intervention methods

Male APP/PS1 mice (APPswe, PSEN1dE9) were purchased from Beijing Huafukang Biotechnology Co., while male C57BL/6J mice were obtained from the Laboratory Animal Center at Guangzhou University of Chinese Medicine. The mice were kept under controlled conditions with a 12-hour light/dark cycle at 25°C and had unrestricted access to food and water. All procedures were conducted in accordance with the Regulations of the Ministry of Science and Technology of the People’s Republic of China on the Management of Laboratory Animals. Additionally, the welfare and testing practices of the Laboratory Animal Center strictly complied with Guangzhou University of Traditional Chinese Medicine’s guidelines for laboratory animal care and ethics. Mice of equivalent age (8 months) and body weight (approximately 30 g each) were randomly assigned to one of five groups (n = 6 for each group): APP/PS1, WT, APP/PS1 + Ginseng (6 g/kg) (The recommended daily intake of ginseng for humans is 0.5 g/kg, with the dose for mice being converted using a body surface area correction factor (Nair and Jacob, 2016)), APP/PS1 + 15 mg/kg Ginsenoside Ro (Ro H), and APP/PS1 + 5 mg/kg Ginsenoside Ro (Ro L) (The dosage was determined based on previous ginsenoside studies (Chu et al., 2014) and preliminary experiments).

The mice received a 1-month gavage treatment. The experimental protocols received approval from the Experimental Animal Center at Guangzhou University of Chinese Medicine, guaranteeing the ethical treatment and welfare of the animals involved (Animal experiment ethical number: 20240111002, date: 11 January 2024).

### 2.3 Sample preparation and HPLC analysis of Ginsenoside Ro in ginseng

To prepare the ginseng sample, 250 g of ginseng slices were ground into coarse powder. The powder was then subjected to reflux extraction with 10 times the amount of ultrapure water, performed three times, each for 60 min. The extract was filtered through gauze, and the filtrate was concentrated using a rotary evaporator (EYELA N-1300, Japan) to achieve a final concentration of 0.55 g/mL. Before HPLC detection, the concentrated extract was thoroughly mixed using a vortex mixer and centrifuged with a high-speed refrigerated centrifuge (12,000 rpm, 5 min). The supernatant was collected for analysis. Additionally, a standard solution of Ginsenoside Ro was prepared at a concentration of 1 mg/mL in methanol and set aside for HPLC detection.

The HPLC analysis was carried out using a SHIMADZU LC-2030C 3D Plus HPLC system (Japan) with LabSolutions software. The mobile phase consisted of solvent A (acetonitrile-formic acid, 100:0.1, v/v) and solvent B (water-formic acid, 100:0.1, v/v). Both ginseng and Ginsenoside Ro samples had an injection volume of 10 µL. The elution program was as follows: from 0 to 20 min, a linear gradient from 5% to 19% A; from 20 to 30 min, a linear gradient from 19% to 30% A; from 30 to 40 min, a linear gradient from 30% to 55% A; and from 40 to 50 min, a linear gradient from 55% to 75% A. The column used was an X-Peonyx-C18 column, with dimensions of 250 mm × 4.6 mm and a particle size of 5 μm (Thermo Fisher, Guangzhou, China).

### 2.4 Preparation of tissue samples

After behavioral assessments, mice from different experimental groups were euthanized with 100 mg/kg Pentobarbital sodium (Liu et al., 2024b; Pang and Laferriere, 2020). Subsequently, decapitation was performed following cardiac perfusion with 0.9% saline. The brains were carefully extracted and fixed in 4% paraformaldehyde (PFA) at 4°C for 24 h. Half of the brain samples were dehydrated using a sucrose gradient, embedded in paraffin, and stored at −80°C for subsequent immunofluorescence (IF) analysis. The remaining brain tissue samples were placed in 1.5 mL tubes and preserved at −80°C for future Western blot analysis and Real-Time Polymerase Chain Reaction.

### 2.5 Open field experiment (OFT) test

The day before the experiment, the mice were transferred to the behavior room to acclimate to the new environment. Initially, the mice were allowed to explore an open area measuring 40 cm × 40 cm × 35 cm without restrictions for 5 min (Shoji and Miyakawa, 2019). During this period, their trajectories were automatically tracked using Shinsoft software (Shanghai Shinsoft Information Technology Co., Ltd.) with a camera. The locomotor abilities of the mice were assessed by measuring the total distance traveled (cm), average speed (cm/s), and time spent in the center (s) (He et al., 2024). The open field was divided into 25 quadrants using the software, with the time spent in the nine central quadrants recorded as the central residence time (Tovote et al., 2015). This measurement is often used to evaluate exploratory behavior, autonomous behavior, and the anxiety-depressive state of mice in a new environment.

### 2.6 Morris water maze (MWM) test

Mice were placed in circular pools divided into four equal-sized sections, with a white circular platform (10 cm in diameter) submerged 1 cm underwater in one of the sections, the location of the hidden platform remained in the same quadrant for all the animals (Othman et al., 2022; Liu et al., 2023b). To locate the platform, the mice were trained daily, navigating using various visual cues on the pool wall within a 60-second timeframe. If a mouse failed to find the platform within 60 s, it was gently guided to the platform and allowed to stay there for 20 s. Each day, the mice underwent four training sessions, entering the water from different points, then go to the area where the platform is located,and this training continued for 5 days (Mifflin et al., 2021). On the sixth day, a formal test was conducted without the platform, requiring the mice to swim for 60 s to find the platform’s original location. Data collected from these 6 days were used to assess the memory and cognitive abilities of the mice.

### 2.7 Thioflavin-T staining

The deparaffinized and rehydrated sections were washed in 1 × PBS for 5 min, repeated 3 times. An immunohistochemistry pen was used to outline the tissue edges to prevent staining solution loss. Pre-prepared Thioflavin-T solution was added to the brain tissue sections, incubated at 37°C for 10 min, protected from light. Sections were transferred to an opaque staining vat with 1× PBS buffer for three 5-minute washes in a light-proof environment. The sections were then washed in 75% ethanol for 5 min each, repeated three times, ensuring complete light protection. Anti-fluorescence quencher sealer with DAPI was added, and the tissue was covered with a coverslip. A fluorescence microscope was used for observation and photography.

### 2.8 Nissl staining

Brain tissues were deparaffinized in TO transparency for two 10-minute sessions, followed by 10 min in anhydrous ethanol, 3 min in 95% ethanol, 3 min in 80% ethanol, 3 min in 70% ethanol, and 2 min in ultrapure water. Processed sections were stained with Nissl staining solution for 10–15 min. Sections were washed twice in ultrapure water, each wash lasting 15 s, followed by 15 s in 95% ethanol and two 2-minute washes in anhydrous ethanol. TO transparency was maintained for 5 min, followed by an additional 5 min in fresh TO transparency. Sections were sealed with neutral dendrimer, resulting in cells showing mottled blue-violet staining.

### 2.9 Immunofluorescence (IF)

After dewaxing and rehydrating the paraffin-embedded mouse brain tissues, 3% hydrogen peroxide solution was applied and incubated at 37°C for 5–10 min. The sections were washed three times with 1 × PBS buffer, each wash lasting 5 min. The sections were then placed in a glass slide holder and immersed in a beaker containing sodium citrate repair solution. They were heated in a microwave oven at high, medium-low, and medium-high settings for 5 min each, then cooled to room temperature. A drop of 5% BSA Immunocontainment Solution was added to the sections and incubated for 1 h at 37°C. After aspirating the serum, primary antibody solution (1:200) in PBS was added and incubated at 4°C overnight. The next day, the sections were washed three times with 1 × PBS (5 min each). Secondary antibodies were incubated at room temperature, protected from light, for 1 h, then washed three times with PBS (5 min each). The slides were sealed with DAPI-containing anti-quenching fluorescent sealer. Sections were observed using the Intelligent Tissue Sections Imaging and Analysis System platform. The primary antibodies used in IF experiments were Bax Rabbit antibody (AF0120), Bcl-2 Rabbit antibody (AF6139), Caspase 3 Rabbit antibody (AF6311), IBA1 Rabbit mAb (A19776), GFAP Rabbit Antibody (PAB32097),Thioflavin-T (T168914), and TUNEL assay kit (GDP1042).

### 2.10 Western blot

Brain homogenized protein samples were lysed with RIPA buffer on ice for 60 min and quantified using a BCA assay kit. The samples were separated by SDS-PAGE polyacrylamide gel electrophoresis, and then the proteins were transferred to 0.22 μm or 0.45 μm PVDF membranes and electrophoresed at 300 mA for 90 min. The PVDF membranes were blocked with 5% BSA for 60 min and incubated with the primary antibody (1:1000) at 4°C overnight. The next day, the PVDF membranes were washed three times with 1× TBST to remove the primary antibody, and then incubated with the enzyme-labeled secondary antibody at room temperature for 2 h. After the addition of ECL, the immunoreactive bands were visualized using the Bio-Rad Gel Doc XR system, and the intensity of the bands was quantified using ImageJ software. The primary antibodies used in this experiment were β-Actin Rabbit Antibody (AF7018), Bax Rabbit antibody (AF0120), Bcl-2 Rabbit antibody (AF6139), Caspase 3 Rabbit antibody (AF6311), IBA1 Rabbit mAb (A19776), GFAP Rabbit Antibody (PAB32097), p-P38 Rabbit Antibody (PC2210), P38 Rabbit Antibody (ET1602-26),ERK Rabbit Antibody (ET1601-29),JNK Rabbit Antibody (ET1601-28),p-ERK Rabbit Antibody (ET1601-13) and p-JNK Rabbit Antibody (ET1601-42).

### 2.11 Real-time polymerase chain reaction (qRT-PCR)

Total RNA was extracted from mouse brain tissue using TRIzol (Accurate Biotechnology, Hunan, China) according to the manufacturer’s instructions. RNA samples were quantified using a micro-UV-Vis spectrophotometer nanophotometer NP80 (IMPLEN, Germany) (Liu et al., 2023b) (Evo M-MLV Reagent Premix). The quantified RNA was reverse transcribed to cDNA according to the instructions and the reaction conditions were 37°C for 15 min, 85°C for 5 s, and 4°C for 5 min (Evo M-MLV Reverse Transcription Kit). Fluorescence was detected using a multimode microplate reader (BioTek, United States) at a wavelength of 460 nm, the CT value of the sample was calculated, and the transcription level was analyzed by the 2-△△CT method (Nahum-Ankonina et al., 2023; Su et al., 2024). The sequences of the primers used in this study are listed in Supplementary Table S1.

### 2.12 ELISA

Following the manufacturer’s protocol, the assay was performed by sequentially adding samples, standards, biotin-conjugated detection antibodies, and horseradish peroxidase (HRP) conjugates to microplates pre-coated with antibodies specific to mouse TNF-α, IL-6, and IL-1β. After appropriate incubation and washing steps, 3,3′,5,5′-tetramethylbenzidine (TMB) substrate was added to initiate colorimetric development. Under HRP catalysis, TMB was converted to a blue product, which subsequently turned yellow upon acidification. The color intensity correlated directly with the concentrations of mouse TNF-α, IL-6, and IL-1β in the samples. Sample concentrations were determined by measuring absorbance at 450 nm using a microplate reader.

### 2.13 Terminal deoxynucleotidyl transferase-mediated dUTP nick end labeling (TUNEL) assay and 4′,6-diamidino-2-phenylindole (DAPI) staining

Paraffin-embedded mouse brain tissue sections were deparaffinized through sequential incubations: environmentally friendly dewaxing solutions I, II, and III (10 min each), followed by three absolute ethanol washes (5 min each), and finally rinsed with distilled water. After gentle drying, tissues were circumscribed using a histochemical pen to create a hydrophobic barrier. Proteinase K working solution was applied dropwise to cover the tissue and incubated at 37°C for 20 min. Slides were washed three times with PBS (pH 7.4) on a destaining shaker (5 min per wash). Permeabilization working solution was then applied to cover the tissue and incubated at room temperature for 20 min, followed by PBS washing. Buffer solution was applied and incubated at room temperature for 10 min.

The TUNEL reaction mixture was prepared by combining TDT enzyme, dUTP, and buffer in a 1:5:50 ratio, with volume adjusted according to tissue size and number of sections. The mixture was applied to cover the tissue, and slides were incubated in a humidified chamber at 37°C for 1 h. Following PBS washing, DAPI staining solution was applied and incubated in darkness at room temperature for 10 min. Slides were then washed with PBS on a destaining shaker and mounted using an anti-fade mounting medium.

Images were captured using a fluorescence microscope (DAPI: ultraviolet excitation, 420 nm emission, blue fluorescence; TMR: 590 nm emission, red fluorescence). Quantification of positive cells was performed using ImageJ software.

### 2.14 Statistical analysis

Statistical analysis is performed using GraphPad Prism eight software (GraphPad Software, San Diego, CA). Error bars in the figures represent the mean ± standard error of the mean (SEM). To determine statistical significance across multiple groups, either one-way or two-way analysis of variance (ANOVA) is employed, followed by Tukey’s *post hoc* test. A p-value of less than 0.05 is considered statistically significant.

## 3 Results

### 3.1 Mouse feeding and handling

**FIGURE 1:**
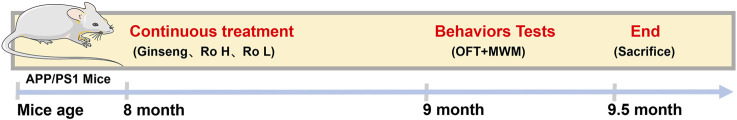
The whole process of mouse breeding and treatment.

Three-month-old male APP/PS1 transgenic AD mice were housed in an SPF-grade animal facility until they reached 8 months of age. At this point, they were treated with gavage administration for 1 month. Following this treatment period, the mice underwent behavioral assays. Upon completion of these assays, samples were collected for subsequent molecular biology testing (The process is shown in Figure 1).

### 3.2 HPLC fingerprint analysis of Ginsenoside Ro in ginseng

**FIGURE 2:**
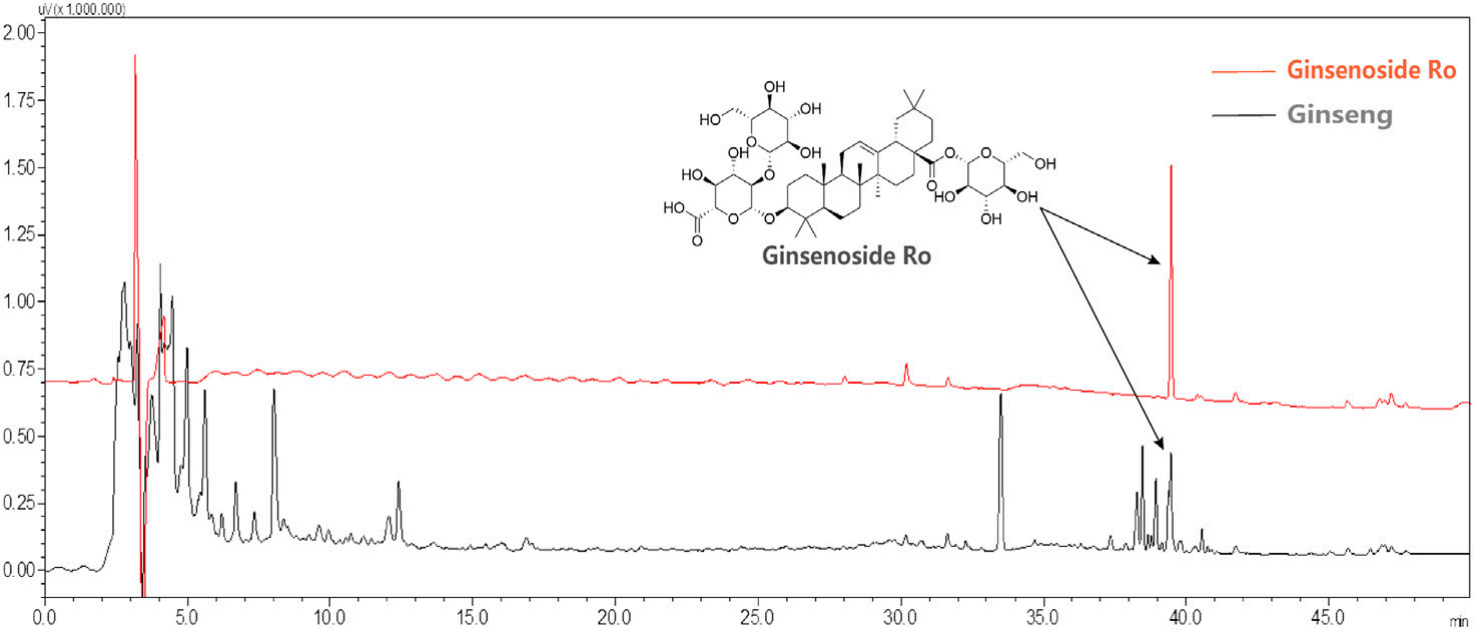
HPLC fingerprint analysis of Ginsenoside Ro in ginseng.

Based on the provided HPLC fingerprint analysis results (Figure 2), Ginsenoside Ro in the ginseng sample was identified through a distinct peak at a retention time of 39.471 min. The analysis was performed at a detection wavelength of 190 nm, which is optimal for ginsenoside detection due to their absorbance characteristics at this wavelength. The chromatogram analysis identifies and separates Ginsenoside Ro from other potential compounds in the sample matrix. This result confirms the presence of Ginsenoside Ro in the ginseng sample and demonstrates the reliability of the HPLC method for identifying such compounds in complex samples.

In summary, the HPLC fingerprint analysis provides an accurate and reliable method for identifying Ginsenoside Ro in ginseng, laying a solid foundation for further research and application of its pharmacological effects.

### 3.3 Ginsenoside Ro improve spatial exploration and anxiety in APP/PS1 mice

**FIGURE 3:**
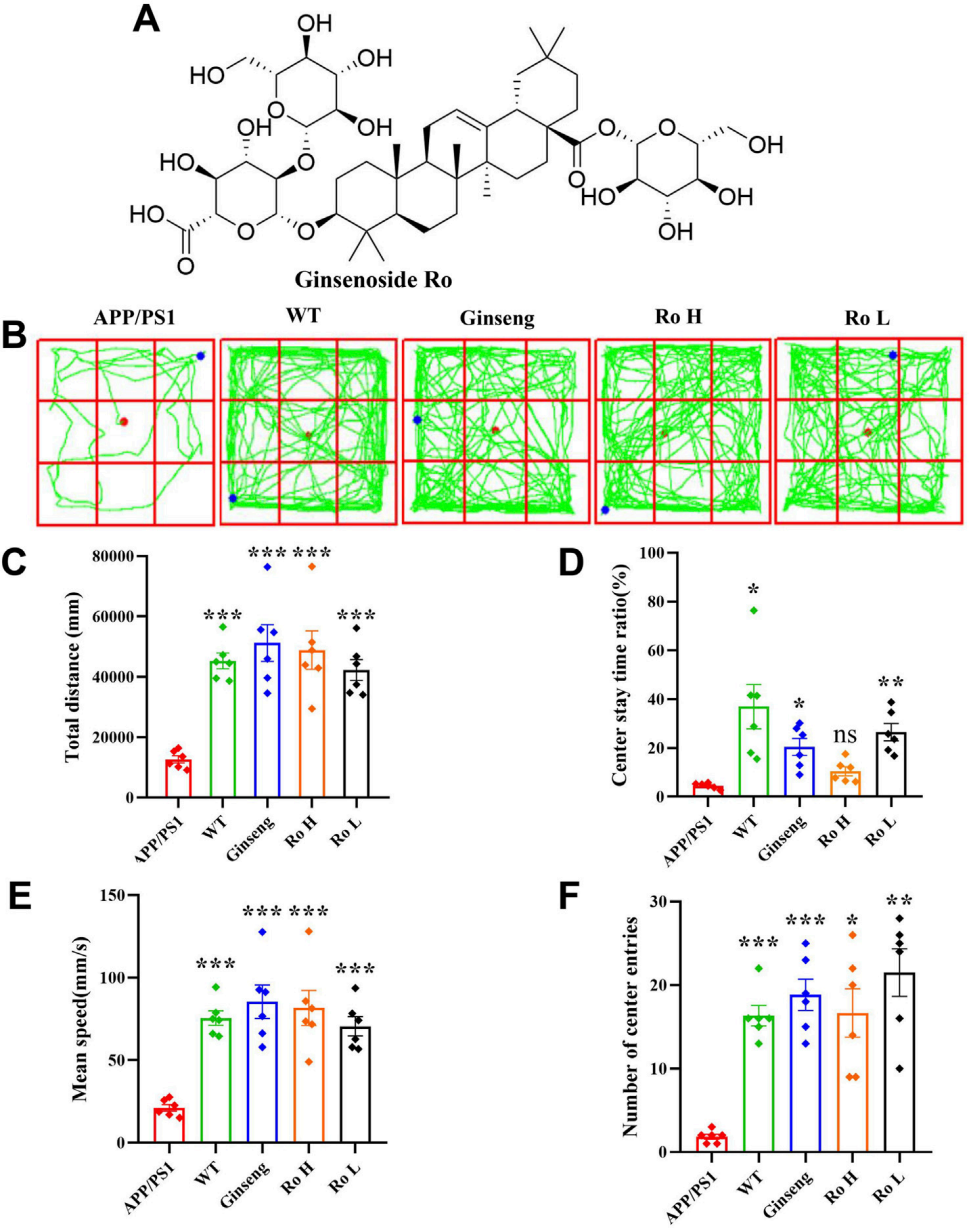
Ginsenoside Ro improve spatial exploration ability and anxiety state in APP/PS1 mice. **(A)** Chemical structural formula of Ginsenoside Ro. **(B)** Diagram of open field trajectory. **(C)** Total distance traveled for locomotion. **(D)** Central residence time. **(E)** Mean speed. **(F)** Number of center entries. Data are expressed as MEAN ± SEM. Statistical significance is indicated as *P < 0.05, **P < 0.01, ***P < 0.001 (n = 6).

The effect of Ginsenoside Ro on spatial exploration ability and anxiety state in APP/PS1 mice was assessed using the open field test (Figure 3). The chemical structural formula of Ginsenoside Ro is shown (Figure 3A). The open field trajectory diagrams (Figure 3B) illustrate that APP/PS1 mice exhibited less exploratory behavior compared to WT mice. Treatments with ginseng, high-dose (Ro H), and low-dose (Ro L) Ginsenoside Ro improved the exploration patterns, making them more similar to WT mice. The total distance traveled for locomotion (Figure 3C) was significantly lower in APP/PS1 mice compared to WT mice, but it was significantly increased in the ginseng, Ro H, and Ro L groups(P < 0.001). The central residence time (Figure 3D) was significantly reduced in APP/PS1 mice compared to WT mice, indicating higher anxiety levels; ginseng (P < 0.05) and Ro L (P < 0.01) treatments significantly increased the central residence time, while Ro H showed an increasing trend without reaching significance. The mean speed (Figure 3E) was significantly lower in APP/PS1 mice compared to WT mice, but it significantly increased with ginseng, Ro H, and Ro L treatments (P < 0.001). The number of center entries (Figure 3F) was also significantly lower in APP/PS1 mice compared to WT mice (P < 0.001), but it significantly increased in the ginseng (P < 0.001), Ro H (P < 0.05), and Ro L (P < 0.01) groups. These results suggest that Ginsenoside Ro, particularly at both doses, significantly improves spatial exploration ability and reduces anxiety in APP/PS1 mice, as evidenced by their improved performance in the open field test.

### 3.4 Ginseng and Ginsenoside Ro improve memory cognitive dysfunction in APP/PS1 mice

**FIGURE 4:**
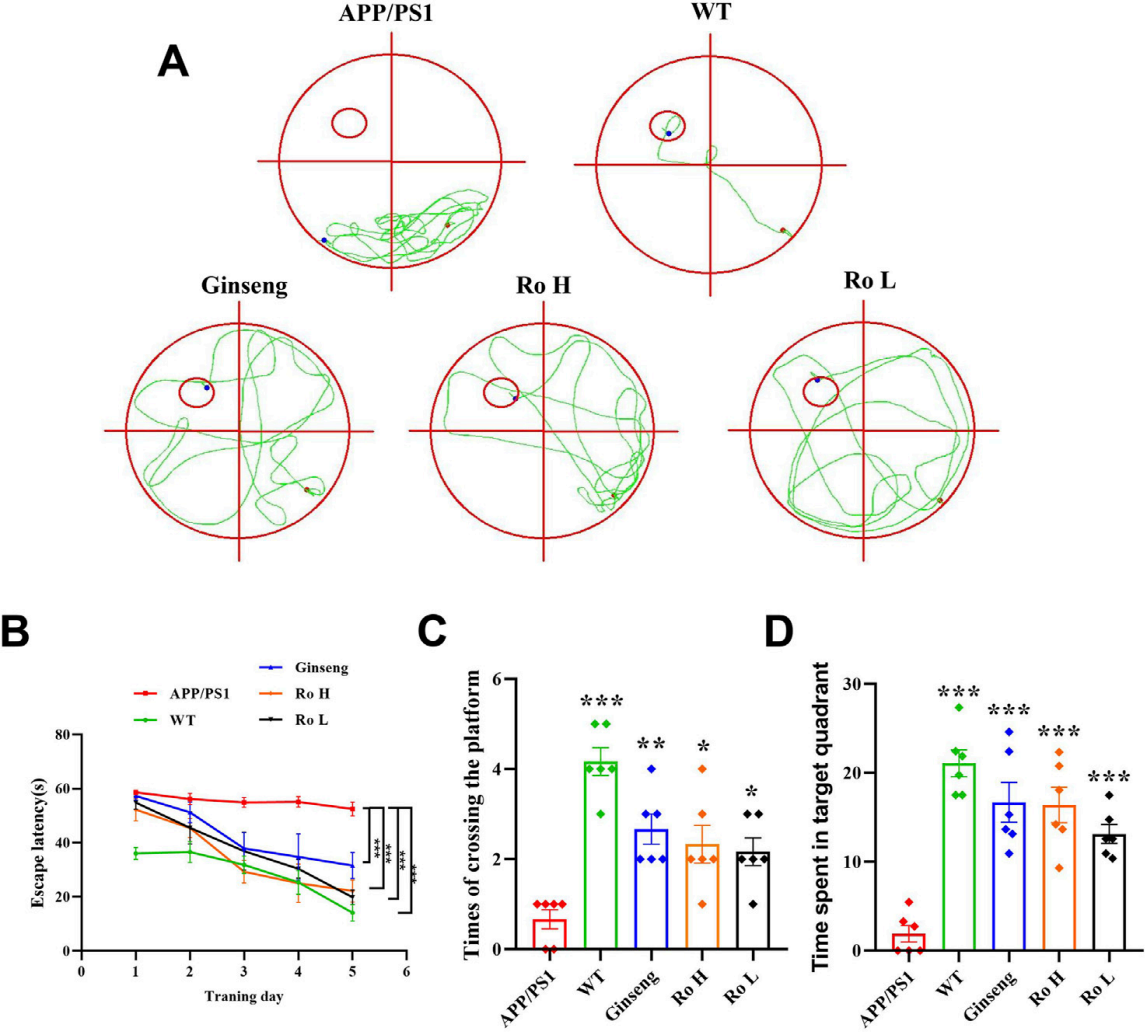
Ginsenoside Ro improves memory and cognitive impairment in APP/PS1 mice. **(A)** Water maze movement trajectory diagram. **(B)** Escape latency. **(C)** Number of platform crossings. **(D)** Time spent in target quadrant. Data are expressed as MEAN ± SEM, and statistical significance is indicated as *P < 0.05, **P < 0.01, ***P < 0.001 (n = 6).

The effect of Ginsenoside Ro on memory and cognitive impairment in APP/PS1 mice was assessed using the MWM test (Figure 4). The water maze movement trajectory diagrams (Figure 4A) show that APP/PS1 mice had a disorganized and lengthy path to find the platform compared to WT mice, which exhibited a direct path. Treatment with ginseng, high-dose (Ro H), and low-dose (Ro L) Ginsenoside Ro improved the path efficiency, resembling that of WT mice. The escape latency graph (Figure 4B) demonstrates that APP/PS1 mice took significantly longer to find the platform over the training days compared to WT mice. Treatments with ginseng, Ro H, and Ro L significantly reduced the escape latency in APP/PS1 mice, indicating improved learning ability (P < 0.001). The histogram of the number of platform crossings (Figure 4C) shows that APP/PS1 mice crossed the platform fewer times compared to WT mice (P < 0.001). Treatments with ginseng, Ro H, and Ro L significantly increased the number of platform crossings (P < 0.05). The histogram of the time spent in the target quadrant (Figure 4D) indicates that APP/PS1 mice spent significantly less time in the target quadrant compared to WT mice. Treatments with ginseng, Ro H, and Ro L significantly increased the time spent in the target quadrant, suggesting improved memory retention (P < 0.001). These results collectively suggest that Ginsenoside Ro significantly improves memory and cognitive function in APP/PS1 mice, as evidenced by better performance in the MWM test.

### 3.5 Efficacy of Ginsenoside Ro in various targets of AD

**FIGURE 5:**
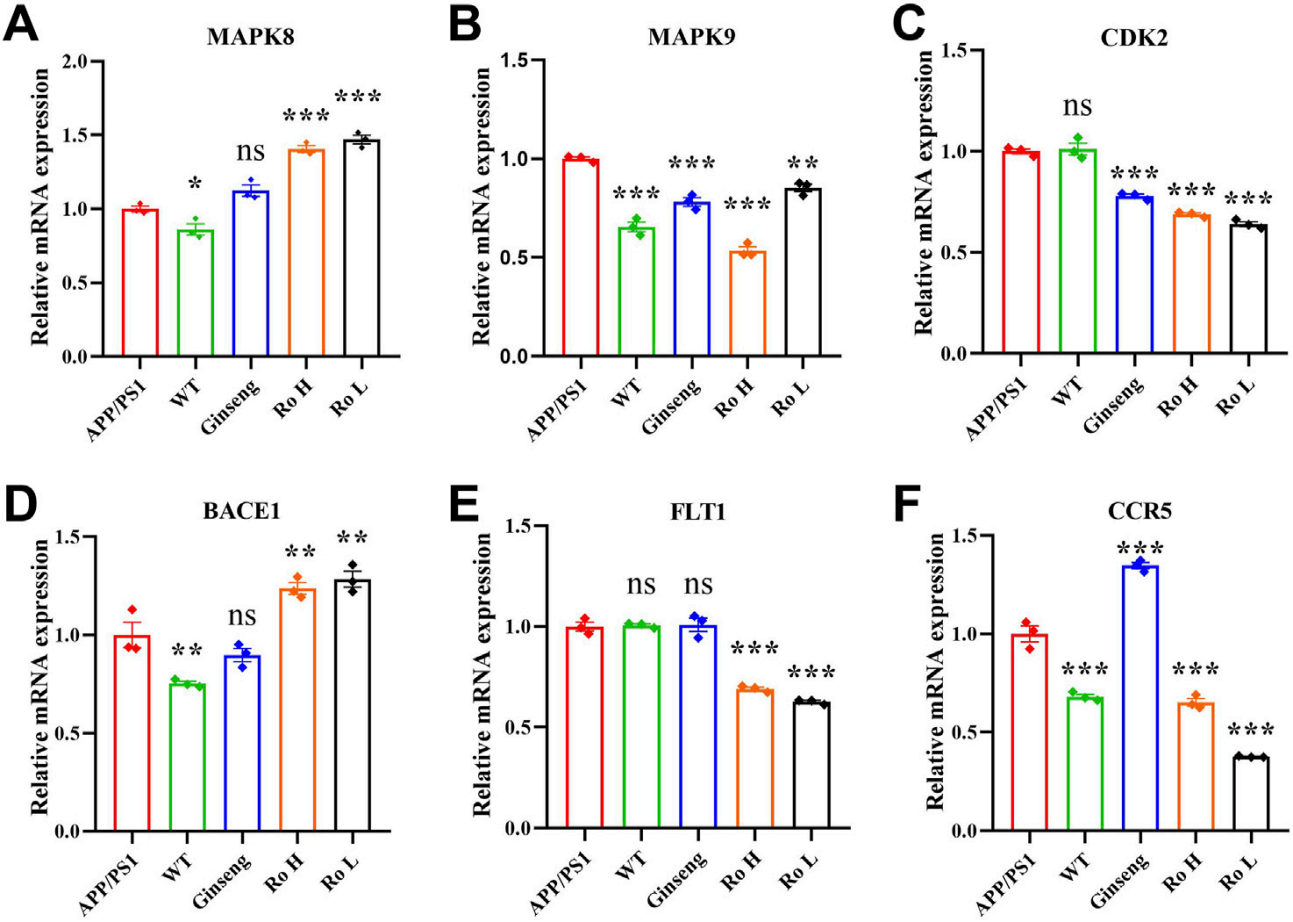
RT q-PCR results of Ginsenoside Ro to modulate the expression of AD-related targets. **(A)** q-PCR results of MAPK8. **(B)** q-PCR results of MAPK9. **(C)** q-PCR results of CDK28. **(D)** q-PCR results of BACE1. **(E)** q-PCR results of FLT1. **(F)** q-PCR results of CCR5. Data are expressed as MEAN ± SEM, and statistical significance is indicated as *P < 0.05, **P < 0.01, ***P < 0.001, (n = 3).

Six AD-related targets were screened in our previous study *in vitro* (Liu et al., 2023a) and this study was extended to *in vivo* models. The effect of Ginsenoside Ro on the expression of these targets was analyzed using RT q-PCR (Figure 5). The results indicate that APP/PS1 mice showed higher relative mRNA expression of MAPK8 compared to WT mice (P < 0.05), and treatments with ginseng, high-dose (Ro H), and low-dose (Ro L) Ginsenoside Ro significantly increased MAPK8 expression (Figure 5A). For MAPK9, APP/PS1 mice had elevated expression compared to WT mice, and both ginseng and Ginsenoside Ro treatments significantly decreased MAPK9 expression, with Ro H (P < 0.001) and Ro L (P < 0.01) showing highly significant reductions (Figure 5B). There was no significant difference in the expression of CDK2 in APP/PS1 mice compared to WT mice., and treatments with ginseng (P < 0.01), Ro H, and Ro L significantly lowered CDK2 expression, with highly significant reductions in the Ro H and Ro L groups (Figure 5C) (P < 0.001). BACE1 expression was elevated in APP/PS1 mice compared to WT mice (P < 0.01), and Ginseng treatment failed to reduce BACE1 expression, whereas Ro H and Ro L treatments increased BACE1 expression (Figure 5D). There was no significant difference in FLT1 expression in APP/PS1 mice compared to WT mice, and Ro H and Ro L treatments significantly reduced FLT1 expression (P < 0.001), whereas ginseng showed no significant change (Figure 5E). CCR5 expression was higher in APP/PS1 mice compared to WT mice, and treatments, including Ro H and Ro L, significantly reduced CCR5 expression (P < 0.001), with highly significant reductions, while the ginseng treatment significantly upregulated (Figure 5F). These results suggest that Ginsenoside Ro, particularly at high doses, can effectively modulate the expression of several AD-related targets, potentially contributing to its therapeutic effects in AD.

### 3.6 Ginsenoside Ro reduce Aβ plaque deposition and ameliorate neuronal apoptosis

**FIGURE 6:**
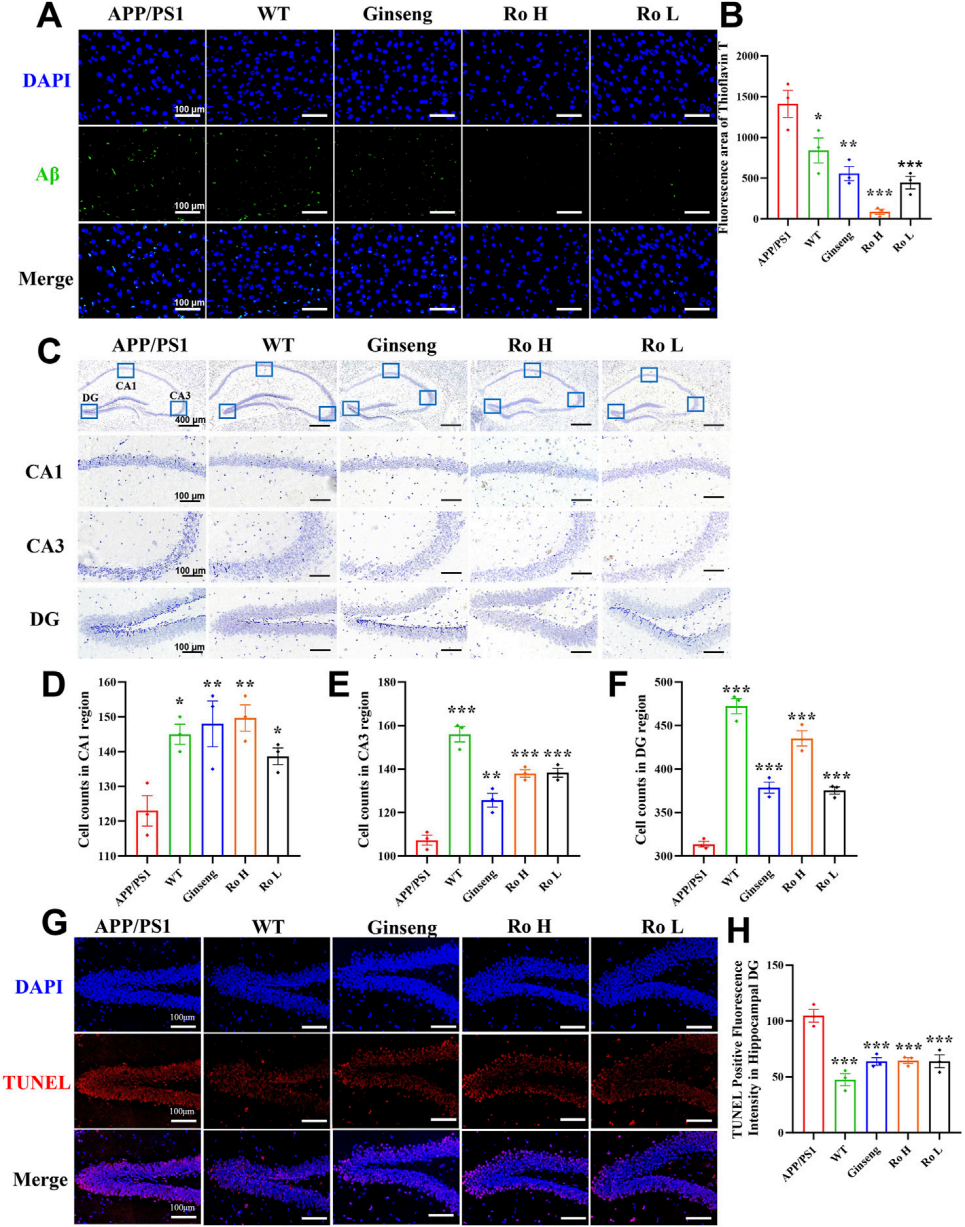
Ginsenoside Ro reduce Aβ deposition and ameliorate neuronal apoptosis in the cerebral cortex of APP/PS1 mice. **(A)** Representative image of thioflavin-T staining fluorescence. **(B)** Histogram of thioflavin-T fluorescence statistics. **(C)** Representative image of Nissl staining in the mouse hippocampal area. **(D)** Histogram of cell counts in the CA1 region. **(E)** Histogram of cell counts in the CA3 region. **(F)** Histogram of cell counts in the DG region. **(G)** Representative graph of fluorescence of TUNEL staining. **(H)** Histogram of fluorescence statistics of TUNEL staining.Data are expressed as MEAN ± SEM. Statistical significance is indicated as *P < 0.05, **P < 0.01, ***P < 0.001 (n = 3).

The effect of Ginsenoside Ro on Aβ deposition and neuronal apoptosis in the cerebral cortex of APP/PS1 mice was analyzed using thioflavin-T staining and Nissl staining (Figure 6). Representative images of thioflavin-T staining (Figure 6A) show that APP/PS1 mice had a significantly higher fluorescence intensity of Aβ deposits compared to the WT and drug-treated groups. The histogram of thioflavin-T fluorescence statistics (Figure 6B) indicates that treatment with ginseng and Ginsenoside Ro significantly reduced Aβ deposition, with the high-dose Ro (Ro H, P < 0.001) group showing a more pronounced effect.

Representative images of Nissl staining in the mouse hippocampal area (Figure 6C) show the neuronal density in different hippocampal regions (CA1, CA3, and DG). The histograms of cell counts in the CA1 (Figure 6D, P < 0.01), CA3 (Figure 6E, P < 0.001), and DG (Figure 6F, P < 0.001) regions reveal that APP/PS1 mice had significantly reduced neuronal counts compared to WT mice. Treatment with ginseng and Ginsenoside Ro significantly ameliorated this reduction, with the high-dose Ro (Ro H) group demonstrating the most substantial improvement in neuronal counts across all regions.

To further confirm the anti-apoptotic effects of ginseng and ginsenoside Ro, we performed TUNEL staining analysis. Results revealed significantly increased TUNEL-positive fluorescence in the hippocampal dentate gyrus (DG) region of APP/PS1 mice compared to both wild-type (WT) and treatment groups (Figure 6G). Quantitative analysis demonstrated that the number of apoptotic cells was significantly lower in the WT group compared to APP/PS1 mice. Moreover, both ginseng and ginsenoside Ro treatment significantly reduced the number of apoptotic cells compared to untreated APP/PS1 mice (Figure 6H, P < 0.001).

These results suggest that ginsenoside Ro is effective in reducing Aβ deposition and ameliorating neuronal apoptosis in the cerebral cortex and hippocampus of APP/PS1 mice, thus potentially attenuating the pathologic effects associated with AD.

### 3.7 Ginsenoside Ro modulates neuronal apoptosis in the hippocampus of APP/PS1 mice

**FIGURE 7:**
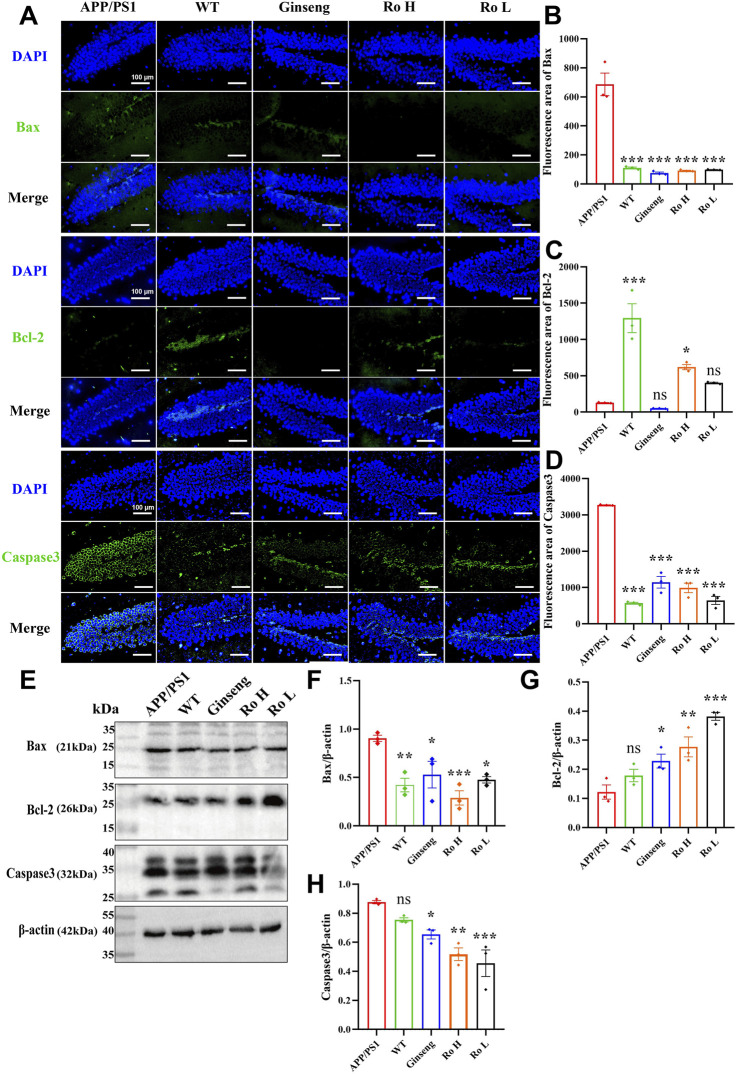
Ginsenoside Ro modulates neuronal apoptosis in APP/PS1 mice. **(A)** Representative images of immunofluorescence staining for Bax, Bcl-2, and Caspase3. **(B)** Histogram of immunofluorescence intensity for Bax. **(C)** Histogram of immunofluorescence intensity for Bcl-2. **(D)** Histogram of immunofluorescence intensity for Caspase3. **(E)** Representative Western blot images for Bax, Bcl-2, and Caspase3. **(F)** Histogram of Western blot data for Bax. **(G)** Histogram of Western blot data for Bcl-2. **(H)** Histogram of Western blot data for Caspase3. Data are expressed as MEAN ± SEM, with statistical significance indicated as *P < 0.05, **P < 0.01, ***P < 0.001 (n = 3).

The effect of Ginsenoside Ro on neuronal apoptosis in APP/PS1 mice was analyzed through immunofluorescence staining and Western blotting for Bax, Bcl-2, and Caspase3 proteins (Figure 7). Representative images of immunofluorescence staining (Figure 7A) show that the fluorescence intensity of pro-apoptotic proteins Bax and Caspase3 was higher in APP/PS1 mice compared to WT and drug-treated groups, while the fluorescence intensity of the anti-apoptotic protein Bcl-2 was higher in the WT and Ro high-dose (Ro H) groups. Histograms of immunofluorescence intensity for Bax (Figure 7B, P < 0.001), Bcl-2 (Figure 7C, P < 0.05), and Caspase3 (Figure 7D, P < 0.001) corroborate these observations, indicating significant reductions in Bax and Caspase3 and an increase in Bcl-2 in the drug-treated groups.

Western blot analysis (Figure 7E) confirmed these findings, showing that the administration of ginseng and Ginsenoside Ro significantly decreased the expression of pro-apoptotic proteins Bax (Figure 7F, P < 0.05) and Caspase3 (Figure 7H, P < 0.01), while increasing the expression of the anti-apoptotic protein Bcl-2 (Figure 7G, P < 0.01). These results suggest that ginseng and Ginsenoside Ro can improve neuronal apoptosis by downregulating pro-apoptotic pathways and upregulating anti-apoptotic mechanisms in APP/PS1 mice.

### 3.8 Ginsenoside Ro ameliorate neuroinflammation in the brains of APP/PS1 mice

**FIGURE 8:**
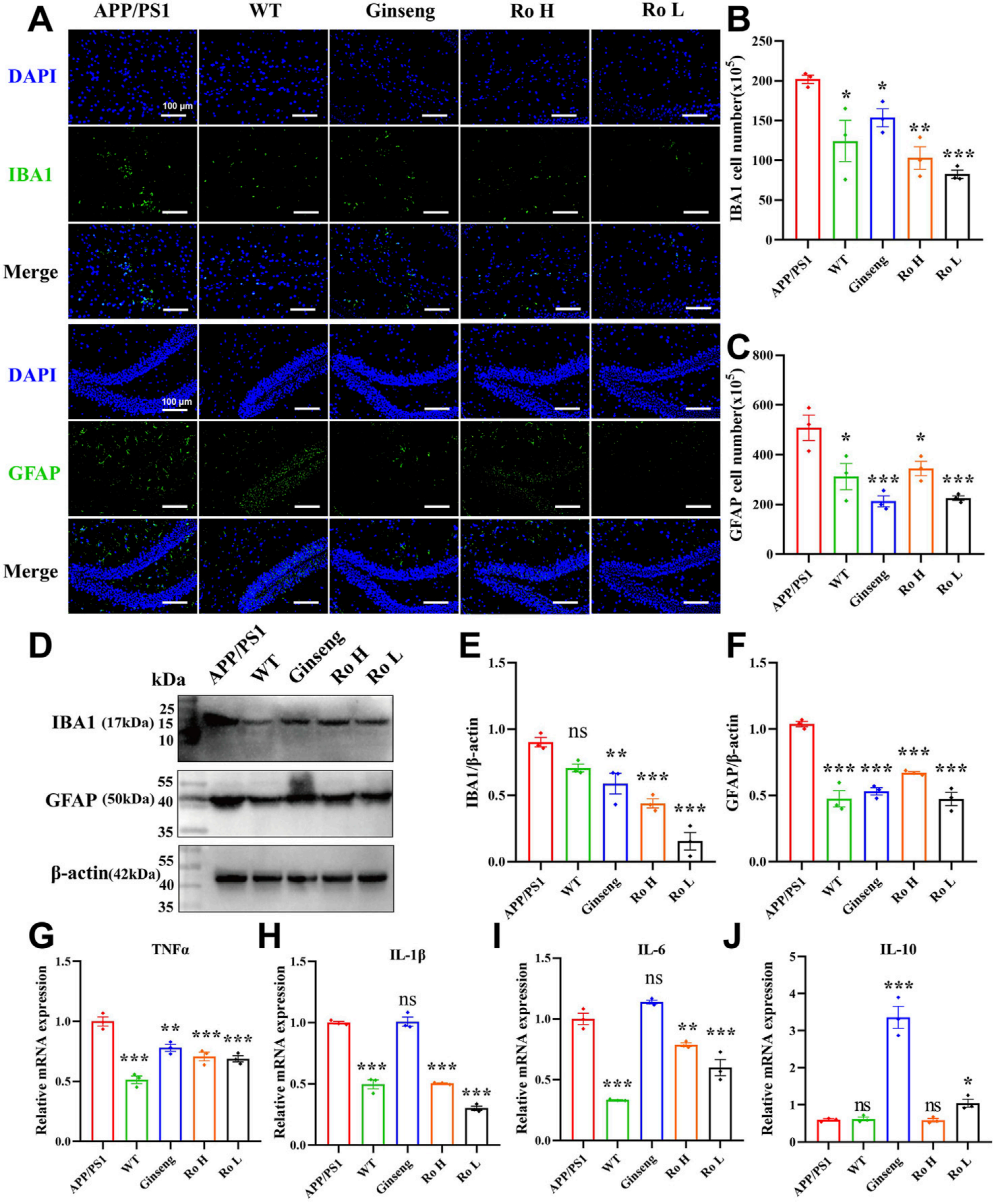
Ginsenoside Ro ameliorates neuroinflammation in the brains of APP/PS1 mice. **(A)** Representative images of IBA1 and GFAP immunofluorescence. **(B)** Histogram of cell activation statistics for IBA1 microglia. **(C)** Histogram of GFAP astrocyte activation statistics. **(D)** Representative images of Western blot for IBA1 and GFAP. **(E)** Histogram of Western blot statistics for IBA1. **(F)** Histogram of Western blot statistics for GFAP. **(G)** Histogram of q-PCR results for TNFα. **(H)** Histogram of q-PCR results for IL-1β. **(I)** Histogram of q-PCR results for IL-6. **(J)** Histogram of q-PCR results for the anti-inflammatory indicator IL-10. Data are expressed as MEAN ± SEM, with statistical significance indicated as *P < 0.05, **P < 0.01, ***P < 0.001 (n = 3).

Neuroinflammation is recognized as a critical factor associated with several disorders leading to cognitive decline and is a key component of AD pathophysiology (Leng and Edison, 2021; Al-Ghraiybah et al., 2022). The effect of Ginsenoside Ro on neuroinflammation in the brains of APP/PS1 mice was analyzed through immunofluorescence staining, Western blotting, and q-PCR (Figure 8). Representative images of IBA1 and GFAP immunofluorescence (Figure 8A) show that APP/PS1 mice had higher activation levels of IBA1-positive microglia and GFAP-positive astrocytes compared to WT mice. Histograms of cell activation statistics for IBA1 (Figure 8B, P < 0.01) and GFAP (Figure 8C, P < 0.05) indicate that drug administration significantly reduced the number of IBA1 and GFAP-activated cells, and no significant dose-dependent effects were seen between the Ginsenoside Ro dosing groups.

Western blot analysis (Figure 8D) confirmed these findings, with the expression levels of IBA1 and GFAP being significantly lower in the drug-treated groups. Histograms of Western blot statistics for IBA1 (Figure 8E, P < 0.001) and GFAP (Figure 8F, P < 0.001) further illustrate the reduction in protein levels. The q-PCR results (Figures 8G–I) demonstrate that Ginsenoside Ro significantly decreased the expression levels of pro-inflammatory factors TNFα, IL-1β, and IL-6, indicating an anti-inflammatory effect. Additionally, q-PCR results for the anti-inflammatory factor IL-10 (Figure 8J) show that ginseng (P < 0.001) and low-dose Ginsenoside Ro (Ro L, P < 0.05) increased IL-10 expression, further supporting the anti-inflammatory properties of the treatments.

To further investigate the anti-inflammatory effects of our treatments, we assessed the expression levels of inflammatory cytokines TNF-α, IL-1β, and IL-6 using ELISA (Supplementary Figure S2). WT mice exhibited significantly lower levels of all three inflammatory markers compared to APP/PS1 mice (P < 0.001). Ginseng treatment significantly reduced the expression of TNF-α (Supplementary Figure S2A, P < 0.05), IL-1β (Supplementary Figure S2B, P < 0.01), and IL-6 (Supplementary Figure S2C, P < 0.001). Similarly, RO H treatment decreased the levels of TNF-α (Supplementary Figure S2A, P < 0.05), IL-1β (Supplementary Figure S2B, P < 0.01), and IL-6 (Supplementary Figure S2C, P < 0.01). RO L treatment also resulted in reduced expression of IL-1β (Supplementary Figure S2B, P < 0.05) and IL-6 (Supplementary Figure S2C, P < 0.05).

These results collectively suggest that Ginsenoside Ro, particularly at high doses, can significantly ameliorate neuroinflammation in the brains of APP/PS1 mice by reducing the activation of microglia and astrocytes, lowering pro-inflammatory factor expression, and increasing anti-inflammatory factor expression.

### 3.9 Mechanism of the effect of Ginsenoside Ro on the MAPK pathway in APP/PS1 mice

**FIGURE 9:**
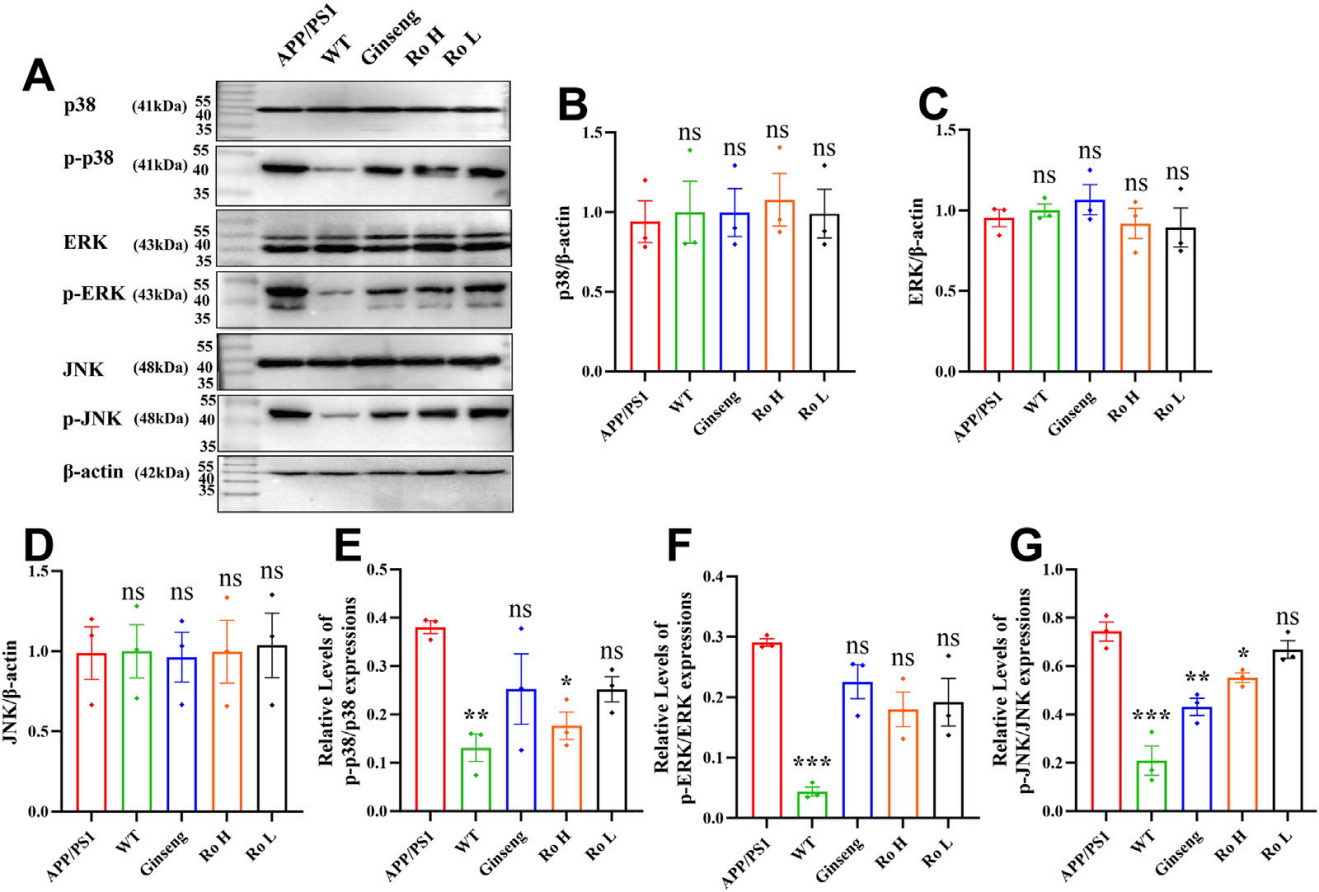
Mechanism of the effect of Ginsenoside Ro on the MAPK pathway in APP/PS1 mice. **(A)** Representative images of Western blot for p-p38, p-ERK, and p-JNK. **(B)** Histogram of Western blot statistics for p38. **(C)** Histogram of Western blot statistics for ERK. **(D)** Histogram of Western blot statistics for JNK. **(E)** Histogram of Western blot statistics for p-p38/p38. **(F)** Histogram of Western blot statistics for p-ERK/ERK. **(G)** Histogram of Western blot statistics for p-JNK/JNK. Data are expressed as MEAN ± SEM, and statistical significance is expressed as *P < 0.05, **P < 0.01, ***P < 0.001 (n = 3)

The impact of ginsenoside Ro on MAPK pathway activation was evaluated by analyzing the phosphorylation levels of p38, ERK, and JNK proteins using Western blot analysis (Figure 9). Quantification of phosphorylated to total protein ratios revealed significantly elevated levels of p-p38, p-ERK, and p-JNK in APP/PS1 mice compared to WT controls. Ro H treatment significantly reduced the levels of p-p38 (Figure 9E, P < 0.05) and p-JNK (Figure 9G, P < 0.05) relative to untreated APP/PS1 mice. Similarly, ginseng administration significantly decreased p-JNK levels compared to APP/PS1 mice (Figure 9G, P < 0.01). While Ro L treatment showed a trend toward reduced p-p38, p-ERK, and p-JNK expression, these changes did not reach statistical significance. Collectively, these findings indicate that ginsenoside Ro, particularly at higher doses, effectively modulates MAPK signaling by reducing the phosphorylation of p38 and JNK, potentially attenuating pathological signaling cascades in APP/PS1 mice.

## 4 Discussion

**FIGURE 10:**
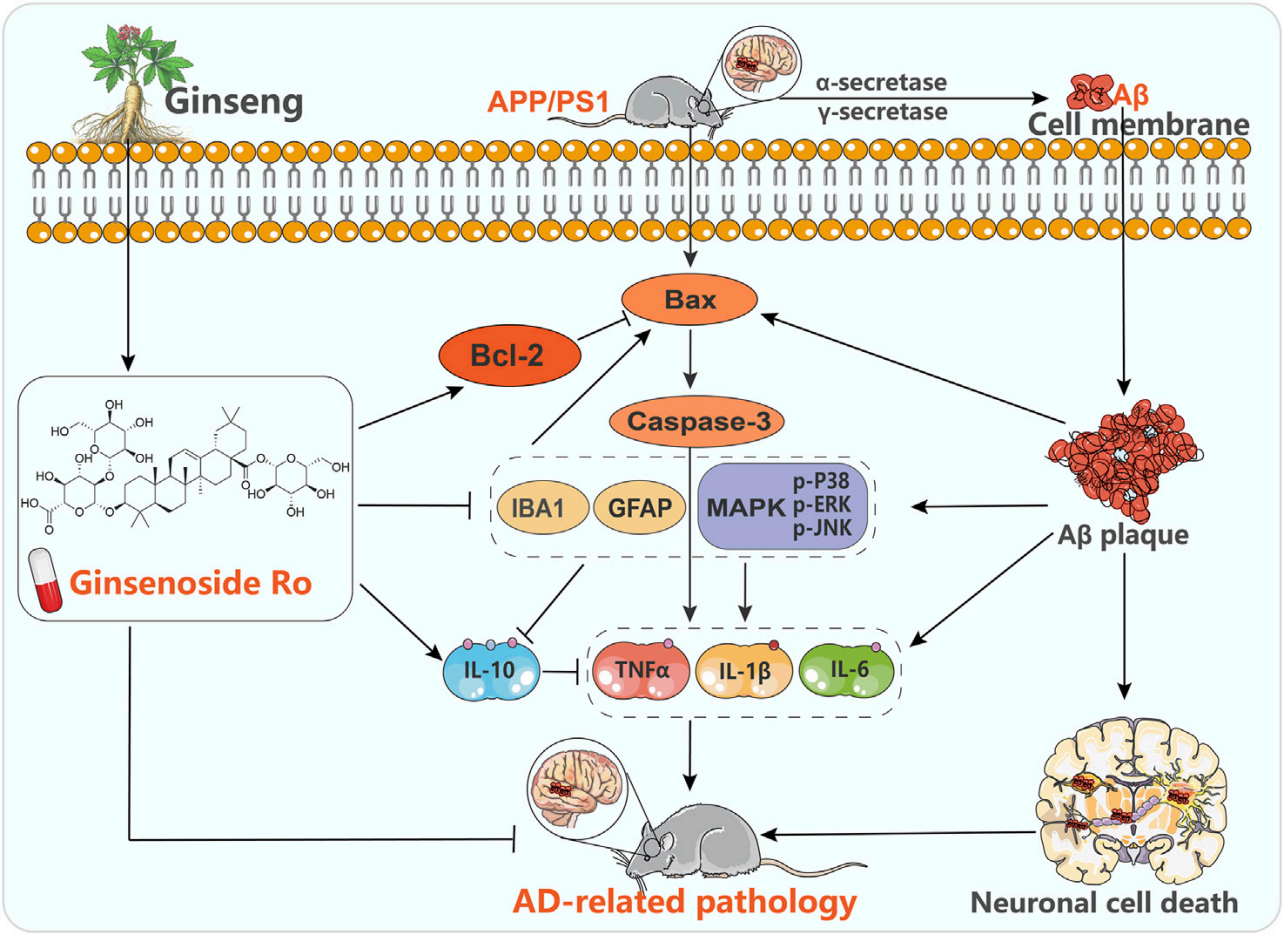
Potential mechanisms of Ginsenoside Ro in the treatment of AD.

This study aimed to investigate the therapeutic potential and underlying mechanisms of Ginsenoside Ro in treating AD using the APP/PS1 transgenic mouse model. The findings demonstrate that Ginsenoside Ro exerts significant neuroprotective effects by ameliorating cognitive deficits, reducing Aβ deposition, and modulating key molecular pathways associated with AD pathogenesis, including neuronal apoptosis and neuroinflammation (Figure 10). These results suggest that Ginsenoside Ro holds promise as a therapeutic agent for AD.

The Morris water maze (MWM) and open field tests (OFT) were employed to assess the cognitive function and anxiety-related behavior of APP/PS1 mice. The results indicate that Ginsenoside Ro significantly improved spatial exploration, learning ability, and memory retention in APP/PS1 mice. Specifically, Ginsenoside Ro treatment reduced escape latency, increased the number of platform crossings, and enhanced the time spent in the target quadrant in the MWM test. These improvements are consistent with previous studies demonstrating the cognitive-enhancing effects of ginsenosides in neurodegenerative models (Rajabian et al., 2019; Huang et al., 2019)

Aβ plaques are a hallmark of AD pathology, and their accumulation is closely linked to neuroinflammation and neuronal death. Our study showed that Ginsenoside Ro significantly reduced Aβ deposition in the brains of APP/PS1 mice, as evidenced by thioflavin-T staining. Additionally, Nissl staining revealed that Ginsenoside Ro ameliorated neuronal loss in the hippocampal regions (CA1, CA3, and DG), further supporting its neuroprotective effects. The immunofluorescence and Western blot analyses indicated that Ginsenoside Ro decreased the expression of pro-apoptotic proteins Bax and Caspase3 while increasing the expression of the anti-apoptotic protein Bcl-2. These findings suggest that Ginsenoside Ro mitigates neuronal apoptosis, which is a critical pathological feature of AD (Yuan et al., 2019).

Previous research has established that microglia and astrocytes are major sources of neuroinflammation through their release of cytokines (Loane and Kumar, 2016; Colonna and Butovsky, 2017; Schober et al., 2022), pro-inflammatory factors, reactive oxygen species, and other immune mediators (Zhao et al., 2024). As key components of the innate immune system, these glial cells play crucial roles in AD pathogenesis (Norris and Kipnis, 2019). While innate immune cells are essential for pathogen elimination and brain homeostasis maintenance in AD, their activation can trigger multiple programmed cell death pathways, including pyroptosis, apoptosis, necroptosis, and panoptosis (Galluzzi et al., 2018; Christgen et al., 2020). The resultant cell death leads to pro-inflammatory cytokine release, which amplifies the innate immune response and facilitates clearance of Aβ plaques and aggregated Tau protein. However, the chronic neuroinflammation stemming from persistent cell death has been implicated in neurodegenerative progression and may contribute to AD exacerbation (Rajesh and Kanneganti, 2022).

Neuroinflammation is a key factor in the progression of AD, and it involves the activation of microglia and astrocytes, leading to the release of pro-inflammatory cytokines (Singh, 2022). Our study demonstrated that Ginsenoside Ro significantly reduced the activation levels of IBA1-positive microglia and GFAP-positive astrocytes in APP/PS1 mice. This was accompanied by decreased expression levels of pro-inflammatory cytokines TNFα, IL-1β, and IL-6, and increased expression of the anti-inflammatory cytokine IL-10. These results are consistent with previous findings that ginsenosides exert anti-inflammatory effects in neurodegenerative diseases (Lu et al., 2022).

MAPKs, including p38, ERK, and JNK, are critical signaling molecules involved in cellular responses to stress and inflammation. The activation of these kinases contributes to inflammation and autoimmunity (Johnson and Lapadat, 2002). Our study showed that Ginsenoside Ro significantly reduced the phosphorylation levels of p38, ERK, and JNK in APP/PS1 mice. This downregulation of the MAPK pathway likely contributes to the observed reduction in neuroinflammation and neuronal apoptosis. The modulation of the MAPK pathway by ginsenosides has been reported in other studies, further supporting our findings (Zhu et al., 2020; Gao et al., 2020). The therapeutic effects of Ginsenoside Ro in APP/PS1 mice can be attributed to its multi-faceted actions on various molecular pathways. By reducing Aβ deposition, Ginsenoside Ro likely mitigates the primary trigger of AD pathology. The reduction in neuroinflammation and neuronal apoptosis further supports the neuroprotective role of Ginsenoside Ro. The involvement of the MAPK pathway provides a mechanistic basis for these effects, as the modulation of this pathway can impact both inflammatory responses and cell survival.

Our findings highlight the potential of Ginsenoside Ro as a therapeutic agent for AD. Given the multi-targeted nature of AD pathology, the ability of Ginsenoside Ro to modulate key pathological features such as Aβ deposition, neuroinflammation, and neuronal apoptosis is particularly advantageous. Future studies should explore the long-term effects of Ginsenoside Ro and its efficacy in combination with other therapeutic agentsAdditionally, clinical trials are warranted to evaluate the safety and efficacy of Ginsenoside Ro in human subjects. Understanding the pharmacokinetics and optimal dosing of Ginsenoside Ro will be crucial for its clinical application. Moreover, exploring the potential synergistic effects of Ginsenoside Ro with other ginsenosides or AD therapies could provide new insights into comprehensive treatment strategies for AD.

## 5 Conclusion

In conclusion, Ginsenoside Ro demonstrates significant neuroprotective effects in the APP/PS1 mouse model of AD. By enhancing cognitive function, reducing Aβ deposition, and modulating neuroinflammation and neuronal apoptosis, Ginsenoside Ro addresses multiple aspects of AD pathology. These findings provide strong support for further investigation of Ginsenoside Ro as a potential therapeutic agent for AD. The modulation of the IBA1/GFAP-MAPK pathway appears to be a key mechanism underlying the beneficial effects of Ginsenoside Ro, offering a promising target for future therapeutic interventions.

## Data Availability

The raw data supporting the conclusions of this article will be made available by the authors, without undue reservation.

## Abbreviations

AD, Alzheimer’s Disease; APP, Amyloid Precursor Protein; APP/PS1: APPswe, PSEN1dE9 transgenic mouse model; Aβ, β-amyloid; BCA, Bicinchoninic Acid; BSA, Bovine Serum Albumin; CA1/CA3/DG, Cornu Ammonis 1/3, Dentate Gyrus (hippocampal regions); C57BL/6J, C57 Black 6/Jackson (mouse strain); DAPI, 4′,6-Diamidino-2-Phenylindole; ERK, Extracellular Signal-Regulated Kinase; FITC, Fluorescein Isothiocyanate; GFAP, Glial Fibrillary Acidic Protein; HPLC, High-Performance Liquid Chromatography; IBA1, Ionized Calcium Binding Adaptor Molecule 1; IL-1β/IL-6/IL-10, Interleukin-1β/6/10; JNK, c-Jun N-terminal Kinase; MAPK, Mitogen-Activated Protein Kinase; MWM, Morris Water Maze; OFT, Open Field Test; PFA, Paraformaldehyde; PBS, Phosphate-Buffered Saline; PVDF, Polyvinylidene Difluoride; qRT-PCR, Quantitative Reverse Transcription Polymerase Chain Reaction; Ro H/Ro L, High/Low Dose Ginsenoside Ro; SDS-PAGE, Sodium Dodecyl Sulfate-Polyacrylamide Gel Electrophoresis; SEM, Standard Error of the Mean; TBST, Tris-Buffered Saline with Tween 20; TNFα, Tumor Necrosis Factor Alpha; TUNEL, Terminal deoxynucleotidyl transferase-mediated dUTP nick end labeling; WT, Wild Type.
