# Supplementary material for: Ginsenoside Ro ameliorates cognitive impairment and neuroinflammation in APP/PS1 mice via the IBA1/GFAP-MAPK signaling pathway
Source: Front Pharmacol. 2025 Feb 24;16:1528590. doi: 10.3389/fphar.2025.1528590 (PMC11891224; doi:10.3389/fphar.2025.1528590)
Supplement: Supplementary file 1 [file DataSheet1.docx]

Supplementary Material

**Contents**

**Fig. S1. WB experimental results in triplicate**

**Fig. S2.** **Histogram of ELSA statistics for TNFα, IL-1β and IL-6.**

**Table S1.** Primer sequences used for q-PCR


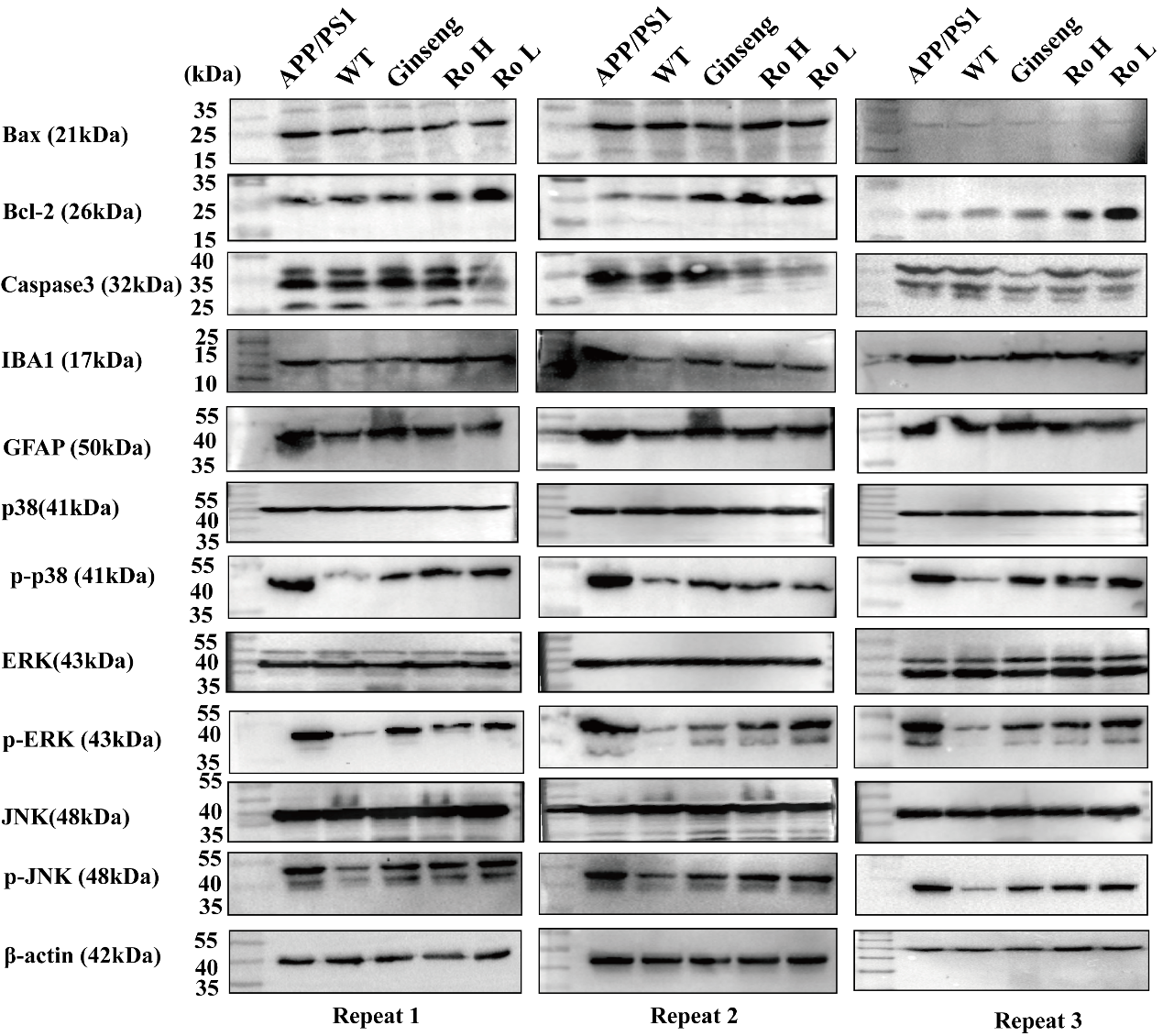


**Fig. S1. WB experimental results in triplicate**

**
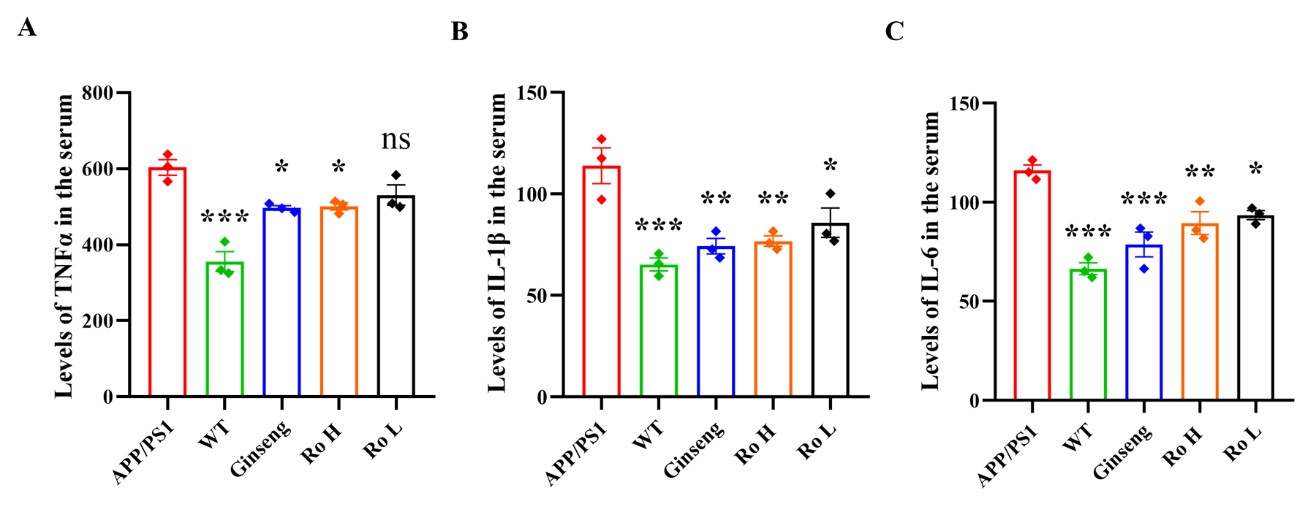
**

**Fig. S2.** **Histogram of ELSA statistics for TNFα, IL-1β and IL-6.**

**Table S1. Primer sequences used for q-PCR**

| Primer name | Forward primer | Reverse primer |
| --- | --- | --- |
| MAPK8  MAPK9  BACE1  CCR5  FLT1  CDK2  TNFα  IL-1β  IL-6  IL10  β-Actin | AGCAGAAGCAAACGTGACAAC  CAGACTGTACCCTCAAGATCCT  CAGTGGGACCACCAACCTTC  TTTTCAAGGGTCAGTTCCGAC  TGGCTCTACGACCTTAGACTG  CCTGCTTATCAATGCAGAGGG  CCCTCACACTCAGATCATCTTCT  GCAACTGTTCCTGAACTCAACT  TAGTCCTTCCTACCCCAATTTCC  GCTCTTACTGACTGGCATGAG  GGCTGTATTCCCCTCCATCG | GCTGCACACACTATTCCTTGAG  ATGCACCCGACAGACCAGA  GCTGCCTTGATGGACTTGAC  GGAAGACCATCATGTTACCCAC  CAGGTTTGACTTGTCTGAGGTT  TGCGGGTCACCATTTCAGC  GCTACGACGTGGGCTACAG  ATCTTTTGGGGTCCGTCAACT  TTGGTCCTTAGCCACTCCTTC  CGCAGCTCTAGGAGCATGTG  CCAGTTGGTAACAATGCCATGT |
